# Optimization of Cementitious Material with Thermal-Activated Lead–Zinc Tailings Based on Response Surface Methodology

**DOI:** 10.3390/ma17122926

**Published:** 2024-06-14

**Authors:** Hang Lin, Ziyi Yin, Su Li

**Affiliations:** School of Resources and Safety Engineering, Central South University, Changsha 410083, China; hanglin@csu.edu.cn (H.L.); 215511016@csu.edu.cn (Z.Y.)

**Keywords:** lead–zinc tailings, thermal activation, cementitious material, optimal material ratio, compression test

## Abstract

The accumulation of lead–zinc tailings will cause a series of problems, including geological disasters and environmental pollution. Efficient secondary utilization of lead–zinc tailings is crucial. In this study, the activity of lead–zinc tailings was stimulated by thermal activation. The optimal thermal activation parameters are a thermal activation temperature of 900 °C and a holding time of 30 min. Based on the response surface methodology, the effect of raw materials content on cementitious material strength was analyzed, and the relational model between cementitious material strength and experimental variables was established. The results show that the sensitivity order of cementitious material strength at 28 days curing age is sand/cement ratio > water/cement ratio > fly ash content > tailing content. According to the relational model, the optimal materials ratio is as follows: tailing/fly ash/cement = 28.99%:14.58%:56.43%, and the sand/binder ratio and water/binder ratio are 1:1 and 0.47, respectively. The corresponding cost is CNY 290.965 per ton, which is the lowest. The strength of cementitious material with these parameters can reach 20 MPa, which meets the requirements of “Technical specification for application of solid waste cementitious material (T/CECS 689-2020)”.

## 1. Introduction

Promoting the pollution prevention of tailing reservoirs is important for sustainable development [1,2,3]. At present, tailings are usually piled directly as waste. It not only occupies a large amount of land but also has a high risk of heavy metal ion leaching, which is easy to cause serious pollution to the surrounding environment. In addition, under extreme weather conditions, tailing reservoirs are prone to damage, resulting in loss of life and property [4]. Therefore, developing comprehensive utilization technology of tailings is of great significance [5].

Due to the presence of SiO_2_, Al_2_O_3_, and other chemical components in tailings, in addition to secondary flotation, using tailings to fabricate cementitious material or other building materials is also effective [6,7,8]. The cementitious activity of tailings is weak and often needs to be stimulated by different methods [9,10,11]. At present, the studies on cementitious materials with tailings mainly focus on iron tailings [12,13,14] and copper tailings [15,16]. Geng Yao et al. [17] studied the change in cementitious activity of iron tailings under mechanical activation and the strength of cement with different iron tailing contents. Jun Shi et al. [18] studied the possibility of using iron tailings and iron tailing ore to replace cement and quartz sand, respectively, and figured out the best substitution ratio of tailings and tailing ore. Hua-Wei Li et al. [19] studied the influence of tailing particle size and the substitution ratio on the strength of cementitious materials. Yannian Zhang et al. [20] used iron tailings, phosphate slag, and lithium slag to fabricate a cementing material that can partially replace cement and showed that the material could improve the strength of the original cement. Felipe Vargas [21] explored the activation of different copper tailings and found the optimal activation parameters of copper tailings used to fabricate cementitious materials. Ahad Barzegar Ghazi et al. [22] regarded copper tailings as supplementary material for cement and found that the addition of tailings can improve the later strength. Alieh Saedi et al. [23,24] explored the possibility of lead–zinc sulfide tailings replacing part of cement and proposed the most suitable substitution ratio. In addition, scholars have also conducted relevant research on the possibility of fabricating cementitious materials from gold, tungsten, and nickel–cobalt tailings [25,26,27,28,29]. Fly ash, a by-product of coal burning, will cause a serious threat to the environment and atmosphere when discharged [30]. Since fly ash contains a large amount of active volcanic ash, it can also be used to participate in the fabrication of cementitious material. On one hand, it can participate in the reaction with calcium oxide to enhance the strength of cement; on the other hand, the particle size of fly ash is small, which can fill the pores inside the cementitious material. There have been many studies on the preparation of cementing materials from fly ash. Based on the previous studies, there are few studies on the preparation of cementitious materials with lead–zinc tailings, let alone the studies on lead–zinc tailing-based cementitious materials incorporated with other solid wastes. This may be due to the environmental impact of heavy metal ions in lead–zinc tailings. In fact, scholars have found that high-temperature calcination can not only decompose carbonaceous minerals such as dolomite to reduce the negative effect on the strength development of cementitious materials, make tailing particles appear loose with an amorphous structure, and enhance cementitious activity but also effectively limit the leaching of heavy metal ions within an environmentally acceptable range [31]. Therefore, conducting research on the fabrication of cementitious materials with thermal-activated lead–zinc tailings and other solid waste is feasible and has practical significance. It can not only reduce the potential harm of lead–zinc tailing storage to the environment and human beings but also enrich the understanding of tailings-based cementitious materials.

In this research, the potential cementitious activity of lead–zinc tailings (LZTs) is stimulated by thermal activation, and the phase change and microstructure of thermal-activated lead–zinc tailings (TA-LZCs) are analyzed by XRD and SEM technology. TA-LZCs and fly ash (FA) were used to substitute part of cement to prepare cementitious material. Taking uniaxial compressive strength as the response value, lead–zinc tailing content, fly ash content, the sand/binder ratio, and the water/binder ratio as design factors, the experiment was carried out by response surface method. The mathematical regression model between the response value and four design factors is established. The influence of a single factor and the interaction of any two factors on the compressive strength of cementitious materials was analyzed, and the sensitivity order of strength to each factor was determined. Based on the strong demand for cementitious materials and the cost of raw materials, the optimal ratio of raw materials was determined.

## 2. Specimen Preparation and Experimental Scheme

### 2.1. Characteristics of Raw Materials

**Figure 1:**
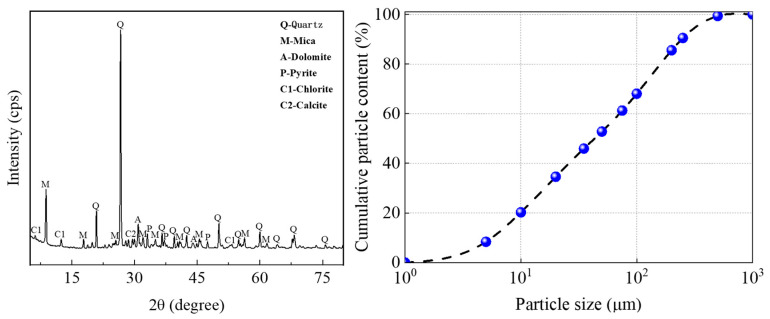
Mineral composition and particle size distribution of lead–zinc tailings.

The raw materials are TA-LZTs, PO425 ordinary Portland cement (OPC), FA, quartz sand, and water. Via X-ray fluorescence (XRF, Bruker S2 PUMA, Bruker, Ettlingen, Germany) analysis, the main chemical component of each raw material is shown in Table 1. It can be concluded that the main chemical component of original LZTs and FA is SiO_2_. As shown in Figure 1, the mineral composition of LZTs was analyzed by X-ray diffraction analysis (XRD, Bruker D8 ADVANCE, Bruker, Germany), and the particle size distribution of LZTs was determined by laser particle size analyzer (Mastersizer 2000 with Hydro2000M, Malvern, Britain). It can be found that the main mineral compositions of LZTs are quartz, mica, dolomite, chlorite, and pyrite. The particle size of lead–zinc tailings is less than 1 mm, and its non-uniformity coefficient Cu = d_60_/d_10_ = 15.297 ≥ 10. The LZTs have good gradation and are easy to fully mix and react to fabricate cementitious material. In addition, the microstructure of LZTs was analyzed by SEM technology (MIRA3 LMH eds: One Max 20, Tescan, Brno, Czech Republic).

### 2.2. Thermal Activation of LZTs

The original LZT powder has obvious crystal characteristics and low cementitious activity. Via high-temperature sintering, the tailings can obtain pozzolanic activity like fly ash. In this study, five levels of thermal activation temperature (850 °C, 900 °C, 950 °C, 1000 °C, and 1050 °C) and three levels of holding time (30 min, 1 h, and 2 h) were designed. According to the full-factorial experiment, a total of 15 different combinations of thermal activation parameters could be obtained. Before thermal activation, lead–zinc tailings are dried at 105 °C for 12 h. The dried lead–zinc tailings are placed in a box resistance furnace (Yiheng SX2-10-12NP, Yiheng, Shanghai, China) with a heating rate of 10 °C/min from room temperature to the specified thermal activation temperature. After thermal activation, the temperature is naturally cooled to room temperature. In addition to measuring the strength of activated lead–zinc tailings, the mineral composition and microstructure of activated lead–zinc tailings under different thermal activation parameters are also analyzed by XRD and SEM to figure out the optimal thermal activation parameters.

### 2.3. Experimental Scheme

In this study, the specimens used to carry out uniaxial loading experiments are divided into two parts, namely the TA-LZTs net-paste specimens and cementitious material with TA-LZTs. TA-LZT net-paste specimens are fabricated from TA-LZTs and water. The procedure included passing the TA-LZT powder through a 65-mesh sieve, adding water to stir evenly with a water/binder ratio of 0.3, and pouring into the mold to fabricate TA-LZT net-paste specimens with size of 40 mm × 40 mm × 40 mm. The fabrication of cementitious material with lead–zinc tailings is based on response surface method design (four factors and three levels). The factors are selected as several important proportional parameters, which are TA-LZT content, FA content, sand/binder ratio, and water/binder ratio. The size of the cementitious material is 40 mm × 40 mm × 40 mm, and it is fabricated from TA-LZTs, FA, quartz sand, and water according to the specified ratio parameters (Table 2). The specimens are successively numbered from 1 to 29, and two parallel specimens are prepared to reduce the experimental error. All specimens are cured under standard curing conditions for 28 days before undergoing uniaxial compression tests. The loading equipment used is a HUALON WHY-300/10 testing machine (Hualong, China) with a maximum range of 300 kN, which meets the experimental requirements. The loading mode is force-controlled, and the loading rate is set to 2.4 kN/s.

## 3. Experimental Results

### 3.1. Analysis of Thermal Activation Experiment

#### 3.1.1. Uniaxial Compressive Strength

**Figure 2:**
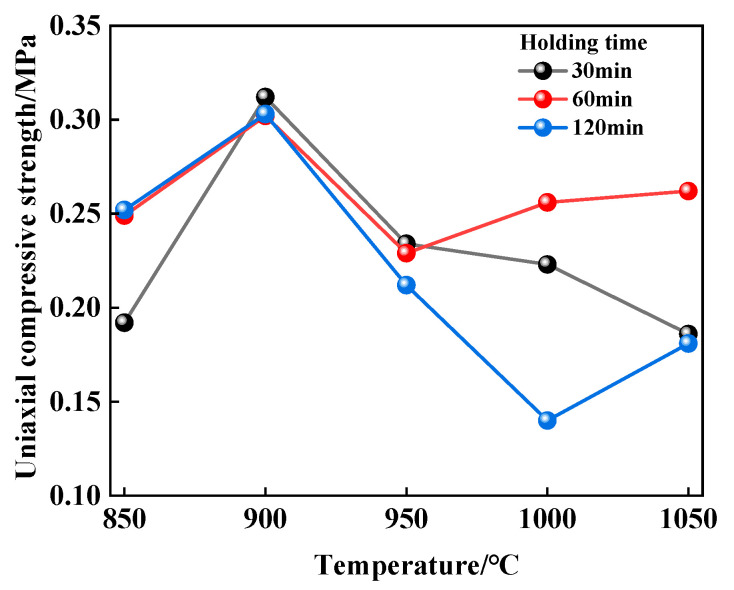
Relationship between thermal activation parameter combinations and uniaxial compressive strength of TA-LZTs net-paste specimens at 28 days curing age.

Figure 2 shows the relationship between the uniaxial compressive strength of TA-LZTs net-paste specimens and thermal activation parameters. Regardless of the holding time, with the increase in thermal activation temperature, the compressive strength generally shows a trend of first increasing and then decreasing and reaches the maximum when the thermal activation temperature is 900 °C. In addition, it is easy to find that when the thermal activation temperature is 900 °C, the strength changes little. The strength reaches the maximum of 0.312 MPa when the holding time is 30 min. Therefore, in order to ensure high strength, the thermal activation temperature should be set to 900 °C. Among all combinations of thermal activation parameters, when the thermal activation temperature is 850 °C, the strength increases with the variation in holding time, and the strength of specimens with holding time of 60 min and 120 min is basically the same. This indicates that at this temperature, thermal activation for 60 min fully stimulates the cementitious activity of LZTs, and more thermal activation time has little positive effect on cementitious performance. When the thermal activation temperature rises to 900 and 950 °C, it can be seen that the strength of the specimen with a holding time of 30 min is the maximum. Therefore, in line with the principle of energy saving and environmental protection, the optimal holding time is set to 30 min. In addition, when the thermal activation temperature is 900 °C, the strength difference in specimens corresponding to three holding times is very small, and when the thermal activation temperature is 950 °C, the strength of the specimen with a holding time of 120 min is significantly lower than that of the other two specimens. This means that when the thermal activation temperature is between 900 and 950 °C, too long of a holding time will reduce the cementitious performance of tailings, which may be caused by the change in crystal structure. This can also be proved by the fact that when the thermal activation temperature is 1000 and 1050 °C, the strength of the specimen with a holding time of 60 min is significantly higher than that of the specimen with a holding time of 120 min.

#### 3.1.2. Analysis of XRD, TG-DSC and SEM

**Figure 3:**
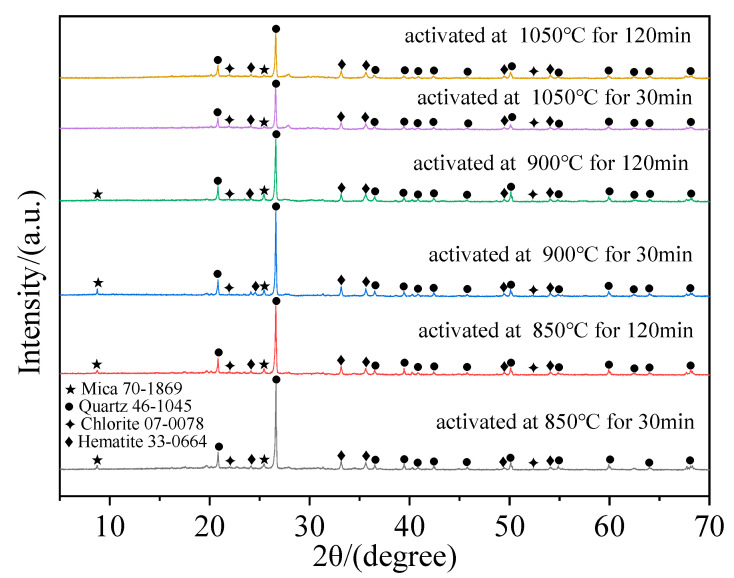
Mineral composition of TA-LZTs activated at different thermal activation parameters.

According to Section 3.1.1, under each holding time, the strength of the specimen shows a trend of first increasing and then decreasing with the variation in thermal activation temperature. Therefore, specimens with thermal activation temperatures of 850, 900, and 1050 °C and holding times of 30 and 120 min are chosen for XRD analysis, as shown in Figure 3. It can be found that when the thermal activation temperature is between 850 and 1050 °C and the holding time is between 30 and 120 min, the main mineral compositions of TA-LZTs are quartz, hematite, mica, and chlorite. Compared with the XRD pattern of original LZTs (Figure 1), after high-temperature thermal activation, the diffraction peaks of dolomite and calcite disappear, and the diffraction peaks of mica and chlorite become less obvious, which is caused by the decomposition of these minerals during thermal activation process. The newly generated diffraction peaks of hematite are mainly derived from the oxidation of pyrite. The relevant chemical equation during thermal activation can be expressed as follows:

(1) $$CaMg(CO_{3}{)}_{2}(Dolomite)\to CaO+MgO+2CO_{2}↑$$

(2) $$4FeS_{2}(Pyrite)+{11O}_{2}\to 2Fe_{2}O_{3}(Hematite)+8SO_{2}↑$$

(3) $$CaCO_{3}(Calcite)\to CaO+CO_{2}↑$$

(4) $${2KAl}_{2}\left({AlSi}_{3}O_{10}\right){\left(OH\right)}_{2}(Mica)\to {6SiO}_{2}+{3Al}_{2}O_{3}+K_{2}O+{2H}_{2}O↑$$

(5) $${Al}_{4}{Si}_{4}O_{10}{\left(OH\right)}_{8}\left(Chlorite\right)\to 2{Al}_{2}O_{3}+4{SiO}_{2}+{4H}_{2}O↑$$

It can be seen from Equation (1) that dolomite decomposed into *CaO*, *MgO*, and *CO*_2_. In Equation (2), pyrite was oxidized to hematite (*Fe*_2_*O*_3_). Calcite will decompose into *CaO* and *CO*_2_ (Equation (3)). From Equation (4), mica (*KAl*_2_*(AlSi*_3_*O*_10_*)(OH)*_2_) in original LZTs mainly decomposes into *SiO*_2_, *Al*_2_*O*_3_, *K*_2_*O*, and *H*_2_*O*. Chlorite (*Al*_4_*Si*_4_*O*_10_*(OH)*_8_) decomposed into *Al*_2_*O*_3_, *SiO*_2,_ and *H*_2_*O* (Equation (5)). When the thermal activation temperature is 900 °C, the diffraction peaks of dolomite and calcite completely disappear. This means that the carbonate minerals are completely decomposed, and the generated *CaO* and *MgO* will react with *H*_2_*O* in the subsequent fabrication of cementitious materials. In addition, the decrease in diffraction peak density of mica and chlorite means that *Si* and *Al* are separated from the original mineral structure, and the activity of LZTs is improved. In addition, under the action of high temperature, the crystal characteristics of quartz also change from a crystalline state to an amorphous state, and its activity is also improved. Compared with XRD patterns of TA-LZTs with the same thermal activation temperature and different holding times, when the thermal activation temperature is 850 °C, the XRD patterns of the two specimens are basically the same. When the holding time is 120 min, the diffraction peak density of quartz is slightly lower at about 20° and 55°, and the diffraction peak density of hematite at 55° is higher. This means that when the thermal activation temperature is 850 °C, LZTs activated for 120 min can obtain better cementitious performance. When the temperature is 900 °C, it can be found that the diffraction peak of mica located at about 10° becomes no longer obvious when the holding time is 120 min. This means that the longer the holding time, the more sufficient the decomposition of mica at this temperature. The hardness of mica is low, but its decomposition products can participate in the formation of minerals with higher strength. However, the strength of TA-LZTs net-paste specimen activated at 900 °C for 120 min is slightly lower than that of TA-LZTs net-paste specimen activated at 900 °C for 30 min. This may be due to the fact that quartz in LZTs changes from an amorphous to crystalline state again under long-time calcination. It can be confirmed by the higher density of crystal diffraction peaks of quartz. When the thermal activation temperature is 1050 °C, it is easy to find that the diffraction peak of mica at about 10° is not obvious, which indicates that even a short holding time can lead to sufficient decomposition of mica at this temperature. In addition, the quartz diffraction peaks are significantly higher (20°, 25°, and 50°) when the holding time is 120 min, implying a higher crystal structure. In general, the increase in thermal activation temperature and holding time can promote decomposition of original minerals and enhance the cementitious activity of LZTs. However, if the thermal activation temperature is too high or the holding time is too long, it will also cause a negative effect on the cementitious performance.

**Figure 4:**
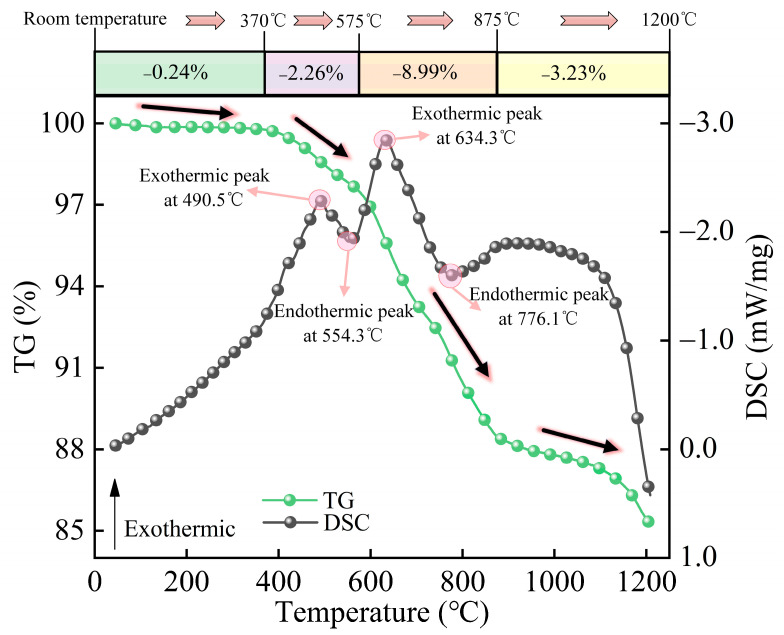
TG-DSC curves of lead–zinc tailings.

To further understand the thermal activation characteristics of lead–zinc tailings, a synchronous thermal analyzer (Netzsch/STA 449 F5 Jupiter, Netzsch, Selb, Germany) was used to perform TG-DSC analysis on lead–zinc tailings, as shown in Figure 4. The results show that as the temperature increases, the weight of lead–zinc tailings gradually decreases, which can be divided into four stages according to the trend of the TG curve. The first stage (from room temperature to 370 °C) mainly corresponds to the evaporation of surface water and crystalline water, with a weight loss of only 0.24%. This is mainly due to the fact that the tailings have already undergone drying before the experiment. In the second stage (from 370 °C to 575 °C), the weight loss rate is 2.26%. At this stage, there exist both exothermic and endothermic peaks. The exothermic peak at 490.5 °C may be caused by the combustion of organic matter in lead–zinc tailings, while the endothermic peak at 554.3 °C may be due to the decomposition of mica and the phase transition of quartz (from α-quartz to β-quartz). Entering the third stage (from 575 °C to 875 °C), the weight of the tailings decreased by 8.99%. It can be inferred that the exothermic peak of 634.3 °C is caused by the oxidation of pyrite into hematite, and the endothermic peak of 776.1 °C is caused by the decomposition of carbonate minerals (dolomite and calcite) in the lead–zinc tailings. The variation in TG and DSC curves during the fourth stage (from 875 °C to 1100 °C) is relatively gentle, indicating that the main change in tailings is melting, which also proves the rationality of setting the thermal activation temperature to 900 °C.

**Figure 5:**
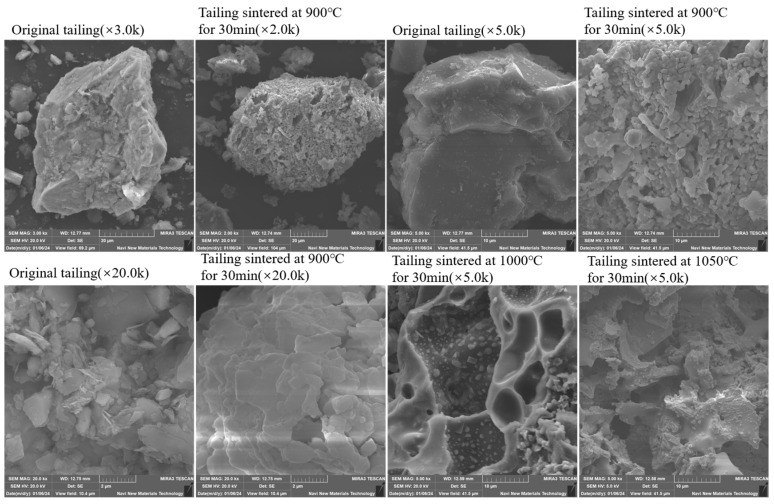
SEM images of original LZTs and TA-LZTs.

The SEM images of LZTs and TA-LZTs are shown in Figure 5. Via comparison, the SEM characteristics of LZTs are bulk particles with smooth surfaces and angular surfaces, showing obvious crystal characteristics. In addition, there are also some small flaky particles. Overall, the microstructure of original LZTs has relatively smooth surface characteristics and compact structural characteristics, and the reaction area is small, which also means that the cementitious activity of original LZTs is relatively weak. The SEM characteristics of LZTs after thermal activation at 900 °C for 30 min show great changes. According to the SEM images at a magnification of 2000 times, it is easy to find that both large bulk particles and small flaked debris particles have loose and porous surface features and the surface of bulk particles is no longer smooth. It can be seen from a magnification of 5000 times that the surface of TA-LZTs is densely distributed with short tentacle-like particles, and the crystal structure is no longer obvious. This is mainly due to the crystal structure changing into a molten state during the high-temperature activation process. This kind of loose structure increases the reaction area and also facilitates the leaching of ions inside the tailings to improve the completion of cementing reaction so as to enhance the cementitious activity of LZTs. By comparing the SEM images magnified at 5000 times of LZTs activated at different temperature for 30 min, it can be seen that TA-LZTs with the highest strength (900 °C for 30 min) have rough and unsmooth surfaces, and the crystal structure is not obvious. The loose and porous surface structure of tailing particles provides a good condition for cementing reaction. For LZTs activated at 1000 and 1050 °C for 30 min, the SEM images show that they have an obvious crystal structure, and the porosity is also significantly reduced, which is not conducive to the leaching of ions.

### 3.2. Analysis of Cementitious Material Based on Response Surface Method

#### 3.2.1. Compressive Strength and Response Surface Model

**Figure 6:**
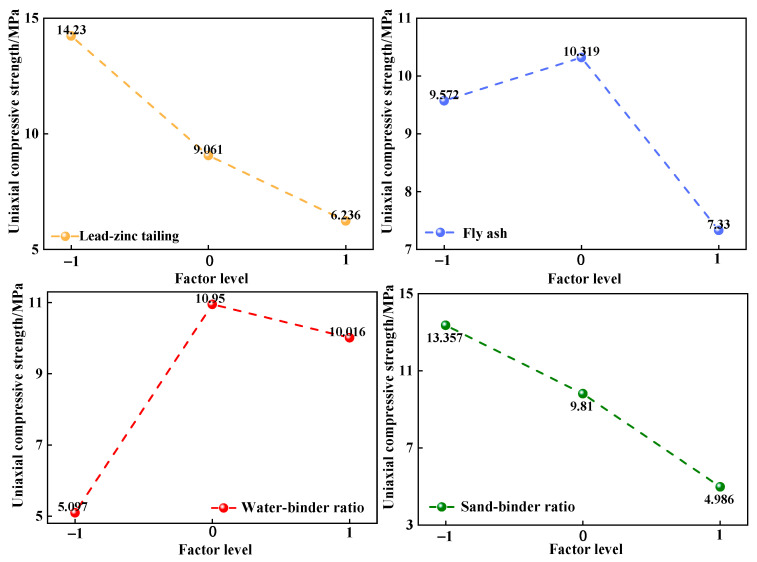
Relationship between proportional parameters and average compressive strength of cementitious materials.

Table 3 shows the raw material ratio based on the response surface method and the corresponding average strength of cementitious material. Based on Table 3, the variation trend of average uniaxial strength under different levels of each proportional parameter is shown in Figure 6. According to the extreme difference (the difference between the maximum value and the minimum value) of each uniaxial strength curve, the sensitivity order of uniaxial compressive strength to four proportional parameters is sand/binder ratio (8.371 MPa) > TA-LZT content (7.994 MPa) > water/binder ratio (5.853 MPa) > FA content (2.989 MPa). From Figure 6, with the increase in TA-LZT content and the sand/binder ratio, the compressive strength curves show a downward trend, indicating that the increase in TA-LZTs and the sand/binder ratio have a weakening effect on the strength of cementitious material. The decrease ranges between 7.994 MPa and 8.371 MPa, respectively, indicating that there is little difference in the influence degree of the two on the strength of cementitious materials. With the increase in the water/binder ratio and FA content, the strength increases first and then decreases. This indicates that the water/binder ratio and FA content should be appropriate; too much or too little will lead to the reduction in cementitious material strength. It should also be pointed out that the water/binder ratio level corresponding to the lowest strength is −1, which is due to the insufficient hydration reaction between raw materials when the water content is not sufficient. In summary, when TA-LZT content is 20%, FA content is 15%, the sand/binder ratio is 1:1, and the water/binder ratio is 0.45, the strength of cementitious material reaches the maximum of 24.263 MPa. When TA-LZT content is 50%, fly ash content is 25%, the sand/binder ratio is 3:1, and the water/binder ratio is 0.35, the strength of cementitious material should be the lowest. Compared with the raw material ratio of group 8 (the cementitious material with the lowest strength listed in Table 3), the sand/binder ratio and water/binder ratio are the same, and the content of TA-LZTs and FA is lower. According to the previous analysis, the strength of cementitious material corresponding to these proportional parameters (TA-LZT content is 50%, fly ash content is 25%, the sand/binder ratio is 3:1, and the water/binder ratio is 0.35) should be lower.

According to the principle of Box–Behnken Design (BBD), Design-Expert software (Design-Expert 13) is used for response surface analysis. Taking the compressive strength of cementitious materials as the response value and taking TA-LZT content, FA content, the sand/binder ratio, and the water/binder ratio as the four design factors, quadratic regression analysis is carried out on the compressive strength and these four proportional parameters, and the relational model between response value and design factors is expressed as follows:

(6) $$\begin{array}{cc} Y & =12.87-4.00A-1.91B-5.00C+2.42D+1.66AB+2.30AC\\ & +0.48AD+1.19BC-0.95BD+0.29CD-0.37A^{2}\\ & -1.76B^{2}-0.72C^{2}-4.34D^{2} \end{array}$$

In Equation (6): *Y*—compressive strength of cementitious material cured for 28 days;

*A*—TA-LZT content;

*B*—FA content;

*C*—sand/binder ratio;

*D*—water/binder ratio.

*AB*, *AC*, *AD*, *BC*, *BD*, and *CD*—interaction terms between TA-LZT content and FA content; TA-LZT content and the sand/binder ratio; TA-LZT content and the water/binder ratio; FA content and the sand/binder ratio; FA content and the water/binder ratio; and the sand/binder ratio and water/binder ratio, respectively.

Table 4 shows the variance results of the relational model, four design factors, and interaction term between each two design factors. It can be seen that the F-value of the relational model is 10.11, and the significance probability *p*-value < 0.0001. When *p*-value < 0.001, it means that the significance of the model is extremely high, indicating that the relational model in this study is extremely significant. From Table 4, the *p*-values of TA-LZT content (*A*) and the sand/binder ratio (*C*) are all less than 0.0001, which indicates that *A* and *C* have significant effects on the compressive strength of cementitious material. The value of the lack of fit was 0.0339 < 0.05. This indicates that the relational model has good fitting ability. In summary, the regression equation is statistically significant. This equation can be used to analyze cementitious material strength and optimize the process parameters.

**Figure 7:**
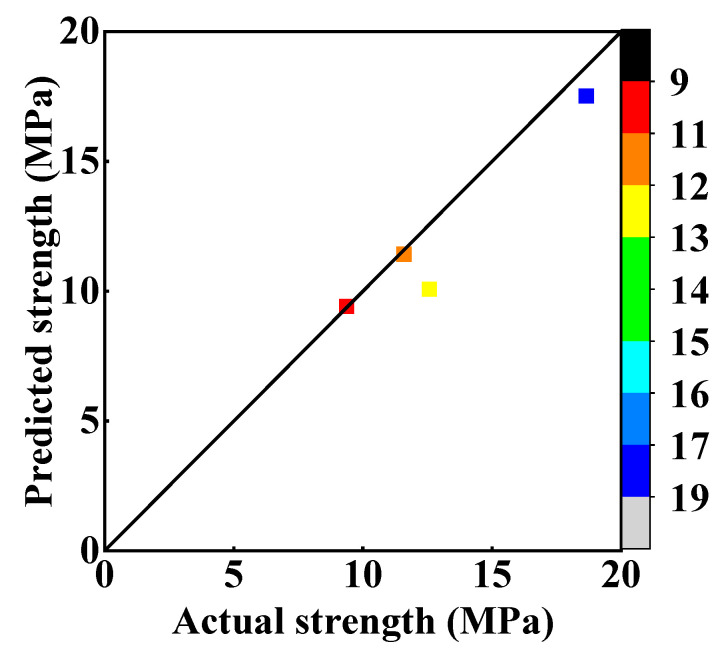
Comparison of predicted strength and actual strength (The squares simultaneously reflect actual strength and predicted strength).

To verify the accuracy of the model, four groups (IDs: 30, 31, 32, and 33) of cementitious materials with different proportional parameters are fabricated to carry out uniaxial compression experiments. The raw material ratio of four cementitious materials and the comparison between experimental values and predicted values are shown in Table 5 and Figure 7. In Figure 7, the comparison of predicted and actual values is very close to the y = x line. This means that the relational model has good reliability and can describe the influence of these four design factors on the uniaxial compressive strength of cementitious material.

#### 3.2.2. Sensitivity Analysis of Interaction between Any Two Factors

**Figure 8:**
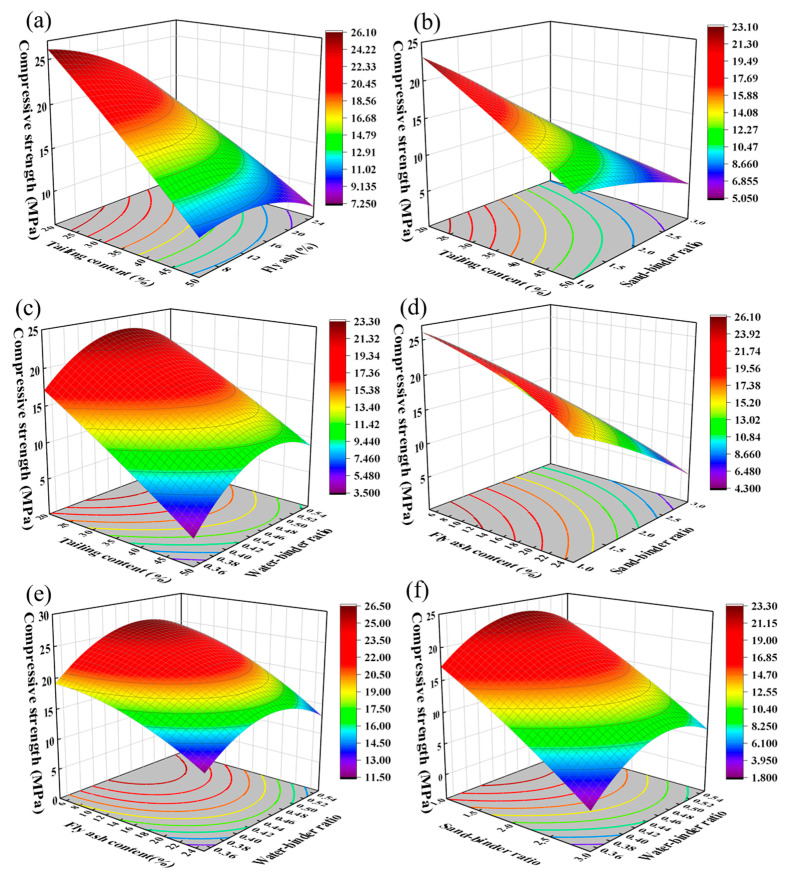
Interaction of any two factors on compressive strength of cementitious materials. (**a**) interaction of tailing content and fly ash. (**b**) interaction of tailing content and sand-binder ratio. (**c**) interaction of tailing content and water-binder ratio. (**d**) interaction of fly ash and sand-binder ratio. (**e**) interaction of fly ash and water-binder ratio. (**f**) interaction of sand-binder ratio and water-binder ratio).

When analyzing the interaction effect of any two factors on the compressive strength of cementitious material, the other two factors are fixed at the level corresponding to their maximum value, respectively. For example, when analyzing the interaction of the water/binder ratio and sand/binder ratio, the factor level of TA-LZT content and FA content are fixed at −1 and 0, respectively. Figure 8a shows the effect of the interaction between TA-LZT content and FA content on the compressive strength of cementitious material. From the variation trend of strength contour lines at the bottom, it can be seen that the strength decreases with the variation in TA-LZTs and FA content. The strength of cementitious material with extremely high contents of lead–zinc tailings (≥50%) and fly ash (≥24%) is less than 7.2 MPa. This indicates that the content of TA-LZTs and FA should be appropriate for fabricating cementitious material that fulfills the strength requirements. With the increase in FA content, the decreasing trend of cementitious material strength with the increase in TA-LZT content slows down. When FA content is 5%, the compressive strength of cementitious material decreases by 2 MPa for every 3.8% increase in TA-LZT content. When FA content is 25%, the compressive strength of cementitious material decreases by 2 MPa for every 6.4% increase in TA-LZT content. With the increase in FA content, the strength of cementitious material decreases slowly first and then rapidly. With the increase in TA-LZT content, the proportion of slow decreasing sections increases with the increase in TA-LZT content.

Figure 8b shows the response surface of interaction between TA-LZT content and the sand/binder ratio on the compressive strength of cementitious materials. The strength of cementitious material decreases with the variation in TA-LZTs and the sand/binder ratio. When one factor level increases, the decreasing trend of cementitious material strength with the increase in the other factor level slows down.

Figure 8c shows the response surface of the interaction between TA-LZT content and the water/binder ratio on the strength of cementitious materials. It can be seen that the strength of cementitious material decreases with the variation in TA-LZT content, and the decreasing trend also slows down with the increase in the water/binder ratio. When the water/binder ratio is in the range of 0.35–0.44, the compressive strength of cementitious materials decreases by 2 Mpa when TA-LZT content increases by 5%. When the water/binder ratio is in the range of 0.44–0.55, the compressive strength of cementitious materials decreases by 2 MPa when TA-LZT content increases by about 7%. In addition, when the TA-LZT content is unchanged, the compressive strength of cementitious material first increases and then decreases with the increase in the water/binder ratio. When the water/binder ratio is between 0.35 and 0.45, the greater the water/binder ratio is, the greater the compressive strength of the cementitious material. As the water/binder ratio increases by 0.02, the compressive strength increases by about 2 MPa. When the water/binder ratio is between 0.45 and 0.55, the larger the water/binder ratio, the smaller the compressive strength of the cementitious material. Moreover, the compressive strength of the cementing material decreases by about 2 MPa as the water/binder ratio increases by 0.05. It can be summarized that a certain amount of water promotes the reaction of raw materials, but an excessive proportion of water will lead to an increase in setting time, which is not conducive to the molding of cementitious materials and weakens the compressive strength.

Figure 8d shows the interaction between FA content and the sand/binder ratio on the compressive strength of cementitious materials. The strength decreases with the increase in FA content and the sand/binder ratio. When FA content is 5%, and the sand/binder ratio is 1:1, the maximum compressive strength can reach 26.1 MPa. According to the variation trend of the bottom contour, for a fixed sand/binder ratio, the strength of cementitious material decreases slowly at first and then rapidly with the increase in FA content. Keeping FA content unchanged, the strength of the cementitious material decreases rapidly with the increase of the sand/binder ratio, and the strength decreases by 2 MPa if the sand/binder ratio increases by about 0.3.

Figure 8e,f, respectively, show the interaction between FA content and the water/binder ratio, sand/binder ratio, and water/binder ratio on the compressive strength of cementitious materials. It can be seen that it is consistent with the interaction law of TA-LZTs and the water/binder ratio. It is worth noting that the curvature of the bottom contour in Figure 8e is larger, which means that the interaction between fly ash and the water/binder ratio is more obvious.

In general, the increase in TA-LZT content and the sand/binder ratio will weaken the strength of cementitious materials, but it will be inhibited by the change in other factors levels. The promotional effect of FA content on the strength of cementitious materials is inhibited by three other factors. Among them, the change in TA-LZT content and the sand/binder ratio slightly inhibit the enhancement effect of FA content on the strength of cementitious materials in the early stage, and the water/binder ratio completely changes the influence law of FA content on the strength of cementitious materials (from increasing–decreasing trend to monotonically decreasing). The influence law of water/binder ratio on cementitious material strength will not be affected by the other three factors. The increase in TA-LZT content and the sand/binder ratio will enhance the strengthening effect of the water/binder ratio on cementitious material strength, while the increase in FA content will inhibit the positive enhancement of the water/binder ratio on cementitious material strength.

#### 3.2.3. Optimization of Raw Material Ratio

According to “T/CECS 689-2020-Technical regulations for the application of solid waste based cementitious materials” [32], the uniaxial strength of cementitious materials with solid waste should not be less than 20 MPa under the curing age of 28 days. For the optimization of the raw material ratio, the premise is to reduce the cost and obtain cementitious materials that meet the strength requirements. In this research, the market price of raw materials is CNY 120/ton for fly ash, CNY 380/ton for cement, and CNY 350/ton for quartz sand. Optimization of the raw material ratio is considered by two different methods. The first is based on actual experimental results and the market price of raw materials and then dividing the cost by the corresponding compressive strength to obtain the price/performance ratio, as shown in Table 6. It is known that if the price/performance ratio is smaller, the utilization rate of raw materials is higher, and the cost is smaller. According to Table 5, the price/performance ratio of group 14 is the smallest and is CNY 12.880/MPa. The corresponding cost of one ton of raw materials is CNY 312.5. The optimal materials ratio obtained by the first method are TA-LZTs/fly ash/cement = 20:15:65, sand/binder ratio is 1:1, water/binder ratio is 0.45, and the corresponding compressive strength is 24.26 MPa.

The second optimization scheme of the material ratio is based on the established response surface model of cementitious material strength. Considering the different prices of raw materials, the preference order of raw material selection is TN-LZTs > FA > quartz sand > cement. This means that TA-LZTs should be preferentially added under the premise of meeting the strength requirements. Since the sand/binder ratio is defined as the mass ratio of quartz sand to binder, the priority of the sand/binder ratio is set to the minimum. In addition, considering that the sum of TA-LZT content, FA content, and cement content is 100%, the priority of TA-LZT content and FA content is set to the highest, and the corresponding cost is the lowest at this time. The range of each material ratio is consistent with Table 2. In addition, the compressive strength of cementitious material should be greater than or equal to 20 MPa under the curing age of 28 days, so the compressive strength should be set to no less than 20 MPa in the model. The specific settings of each factor are shown in Table 7. The optimal material ratio of cementitious material that meets the strength requirement is TA-LZT content/FA content/cement content = 28.99:14.58:56.43, the sand/binder ratio is 1:1, the water/binder ratio is 0.47, and the compressive strength is predicted to be 20 MPa according to Equation (6). The cost of raw materials used in one ton is CNY 290.965/ton, and the price/performance ratio is CNY 14.548/MPa. 

## 4. Conclusions

(1) The mineral composition of lead–zinc tailings contain quartz, mica, pyrite, and chlorite. The optimal combination of thermal activation parameters is activated at 900 °C for 30 min. After thermal activation, the diffraction peak of mica and chlorite is not obvious. The diffraction peaks of dolomite, pyrite, and calcite disappear, and the diffraction peaks of hematite appear. The surface of lead–zinc tailing particles activated at 900 °C for 30 min has a loose and porous structure, and the reaction area increases, which is conducive to the cementing reaction and the leaching of free ions inside the particles.
(2) The sensitivity order of cementitious material strength to four factors is sand/binder ratio > TA-LZT content > water/binder ratio > FA content. The regression model considering cementitious material strength (Y) and TA-LZT content (A), FA content (B), the sand/binder ratio (C) and the water/binder ratio (D) was established. According to interaction analysis, the weakening effect of TA-LZT content and the sand/binder ratio on cementitious material strength would be inhibited by three other factors. The positive strengthening effect of FA content on cementitious material strength is inhibited by three other factors. The enhancement of the water/binder ratio on cementitious material strength is enhanced by TA-LZT content and the sand/binder ratio but inhibited by FA content.
(3) Based on the cost and strength requirement, two different methods are used to optimize the raw material ratio. The optimal material ratio obtained by the first method is TA-LZT content/FA content/cement content = 20%:15%:65%, the sand/binder ratio is 1:1, and the water/binder ratio is 0.45. The cost of one ton of raw materials used is CNY 312.5, and the corresponding price/performance ratio is CNY 12.880/MPa. The optimal ratio obtained by the second method is as follows: TA-LZT content/FA content/cement content = 28.99%:14.58%:56.43%, the sand/binder ratio is 1:1, and the water/binder ratio is 0.47. The cost of one ton of raw materials used is CNY 290.965, and the corresponding price/performance ratio is CNY 14.548 /MPa.

## Figures and Tables

**Table 1:** The main chemical component of raw materials.

Materials	Chemical Component (%)										
	Fe_2_O_3_	SiO_2_	Al_2_O_3_	MgO	CaO	K_2_O	MnO_2_	TiO_2_	Na_2_O	ZnO	Others
LZTs	14.15	48.17	10.79	4.14	4.20	3.01	0.73	0.31	0.46	0.49	13.55
OPC	14.24	2.46	5.41	1.80	52.84	0.89	/	/	0.28	/	/
FA	48.80	4.87	26.26	1.84	4.95	2.00	/	/	1.67	/	/

**Table 2:** Response surface experimental factors and levels.

ID	Factors	Levels		
		−1	0	1
*A*	TA-LZTs	20%	35%	50%
*B*	FA	5%	15%	25%
*C*	Sand/binder ratio	1:1	2:1	3:1
*D*	Water/binder ratio	0.35	0.45	0.55

**Table 3:** Proportional parameters and corresponding average compressive strength of cementitious materials based on response surface method.

ID	TA-LZTs	FA	Cement	Sand/Binder Ratio	Water/Binder Ratio	Strength/MPa
1	50%	5%	45%	2: 1	0.45	5.486
2	35%	5%	60%	1:1	0.45	19.416
3	35%	5%	60%	2:1	0.35	4.185
4	35%	15%	50%	2:1	0.45	11.604
5	20%	25%	55%	2:1	0.45	11.171
6	20%	15%	65%	2:1	0.55	12.953
7	35%	15%	50%	3:1	0.55	6.664
8	35%	15%	50%	3:1	0.35	2.499
9	35%	15%	50%	2:1	0.45	12.485
10	35%	15%	50%	2:1	0.45	14.095
11	35%	25%	40%	3:1	0.45	3.899
12	35%	5%	60%	3:1	0.45	4.818
13	50%	15%	45%	1:1	0.45	13.508
14	20%	15%	65%	1:1	0.45	24.263
15	20%	5%	75%	2:1	0.45	19.493
16	50%	25%	25%	2:1	0.45	3.792
17	35%	25%	40%	2:1	0.55	8.789
18	35%	15%	50%	1:1	0.55	11.016
19	35%	15%	50%	1:1	0.35	8.026
20	35%	25%	40%	2:1	0.35	2.59
21	35%	15%	50%	2:1	0.45	13.634
22	50%	15%	35%	2:1	0.35	2.570
23	35%	5%	60%	2:1	0.55	13.859
24	35%	15%	50%	2:1	0.45	12.537
25	50%	15%	35%	2:1	0.55	6.812
26	50%	15%	35%	3:1	0.45	5.250
27	35%	25%	40%	1:1	0.45	13.739
28	20%	15%	65%	3:1	0.45	6.787
29	20%	15%	65%	2:1	0.35	10.714

**Table 4:** Variance analysis for the regression model.

Source of Variance	Quadratic Sum	Freedom	Mean Square	F-Value	*p*-Value
Model	780.82	14	55.77	10.11	<0.0001
*A*	192.35	1	192.35	34.87	<0.0001
*B*	43.83	1	43.83	7.94	0.0137
*C*	300.51	1	300.51	54.47	<0.0001
*D*	70.49	1	70.49	12.78	0.0030
*AB*	10.98	1	10.98	1.99	0.1801
*AC*	21.24	1	21.24	3.85	0.0699
*AD*	0.92	1	0.92	0.17	0.6888
*BC*	5.66	1	5.66	1.03	0.3283
*BD*	3.65	1	3.65	0.66	0.4297
*CD*	0.34	1	0.34	0.063	0.8062
*A* ^2^	0.88	1	0.88	0.16	0.6956
*B* ^2^	20.10	1	20.10	3.64	0.0770
*C* ^2^	3.39	1	3.39	0.61	0.4464
*D* ^2^	122.21	1	122.21	22.15	0.0003
Residual error	77.24	14	5.52		
Lack of fit	73.31	10	7.33	7.46	0.0339
Absolute error	3.93	4	0.98		
Total dispersion	858.06	28		10.11	

**Table 5:** Residual value of compressive strength in validation group.

ID	*A*	*B*	*C*	*D*	Actual Value (MPa)	Predicted Value (MPa)	Residual Value (MPa)
30	25%	10%	1.5:1	0.4	18.640	17.532	1.108
31	25%	20%	2.5:1	0.5	12.562	10.080	2.482
32	40%	10%	1.5:1	0.4	11.584	11.437	0.147
33	40%	20%	2.5:1	0.5	9.362	9.427	−0.065

**Table 6:** Price/performance ratio calculated based on experimental groups.

ID	Total Cost (CNY/ton)	Price/Performance Ratio (CNY/MPa)	ID	Total Cost (CNY/ton)	Price/Performance Ratio (CNY/MPa)
1	299	54.502	15	337	17.288
2	297	15.297	16	281.667	74.279
3	318	75.986	17	300.667	34.209
4	309.333	26.657	18	284	25.781
5	319.667	28.616	19	284	35.385
6	328.333	25.348	20	300.667	116.088
7	322	48.319	21	309.333	22.688
8	322	128.852	22	290.333	112.970
9	309.333	24.776	23	318	22.945
10	309.333	21.946	24	309.333	24.674
11	315.5	80.918	25	290.333	42.621
12	328.5	68.182	26	307.75	58.619
13	261.429	19.354	27	271	19.725
14	312.5	12.880	28	336.25	49.543
			29	328.333	30.645

**Table 7:** The setting of factor levels and compressive strength.

Factor	Priority	Minimum	Maximum
TA-LZT content (*A*)	Highest	20	50
FA content (*B*)	Highest	5	25
Sand/binder ratio (*C*)	Lowest	1	3
Water/binder ratio (*D*)	In the range	0.35	0.55
Compressive strength	In the range	20	/

## Data Availability

The data used to support the findings of this study are available from the corresponding author upon request.
